# mTOR and autophagy pathways are dysregulated in murine and human models of Schaaf-Yang syndrome

**DOI:** 10.1038/s41598-019-52287-2

**Published:** 2019-11-04

**Authors:** Emeline Crutcher, Rituraj Pal, Fatemeh Naini, Ping Zhang, Magdalena Laugsch, Jean Kim, Aleksandar Bajic, Christian P. Schaaf

**Affiliations:** 1 Translational Biology and Molecular Medicine, Baylor College of Medicine, Houston, TX 77030 USA; 2 Department of Molecular and Human Genetics, Baylor College of Medicine, Houston, TX 77030 USA; 3 Jan and Dan Duncan Neurological Research Institute, Texas Children’s Hospital, Houston, TX 77030 USA; 4 Human Neural Differentiation Core, Jan and Dan Duncan Neurological Research Institute, Texas Children’s Hospital, Houston, TX 77030 USA; 5 Department of Molecular and Cellular Biology, Stem Cells and Regenerative Medicine Center, Center for Cell and Gene Therapy, Baylor College of Medicine, Houston, TX 77030 USA; 6 Human Stem Cell Core, Advanced Technology Cores, Baylor College of Medicine, Houston, TX 77030 USA; 7 Institute of Human Genetics, Heidelberg University, 69120 Heidelberg, Germany

**Keywords:** Mechanisms of disease, Autism spectrum disorders, Stem-cell differentiation

## Abstract

*MAGEL2* is a maternally imprinted, paternally expressed gene, located in the Prader-Willi region of human chromosome 15. Pathogenic variants in the paternal copy of *MAGEL2* cause Schaaf-Yang syndrome (SHFYNG), a neurodevelopmental disorder related to Prader-Willi syndrome (PWS). Patients with SHFYNG, like PWS, manifest neonatal hypotonia, feeding difficulties, hypogonadism, intellectual disability and sleep apnea. However, individuals with SHFYNG have joint contractures, greater cognitive impairment, and higher prevalence of autism than seen in PWS. Additionally, SHFYNG is associated with a lower prevalence of hyperphagia and obesity than PWS. Previous studies have shown that truncating variants in *MAGEL2* lead to SHFYNG. However, the molecular pathways involved in manifestation of the SHFYNG disease phenotype are still unknown. Here we show that a *Magel2* null mouse model and fibroblast cell lines from individuals with SHFYNG exhibit increased expression of mammalian target of rapamycin (mTOR) and decreased autophagy. Additionally, we show that SHFYNG induced pluripotent stem cell (iPSC)-derived neurons exhibit impaired dendrite formation. Alterations in SHFYNG patient fibroblast lines and iPSC-derived neurons are rescued by treatment with the mTOR inhibitor rapamycin. Collectively, our findings identify mTOR as a potential target for the development of pharmacological treatments for SHFYNG.

## Introduction

*MAGEL2* is a maternally imprinted, paternally expressed, single exon gene, located in the Prader-Willi region of human chromosome 15. Nonsense and frameshift mutations of the paternally inherited copy of *MAGEL2* cause Schaaf-Yang syndrome (SHFYNG, MIM 615547), a neurodevelopmental disorder similar to Prader-Willi syndrome (PWS, MIM 176270)^1^. Individuals with Schaaf-Yang syndrome (SHFYNG), like PWS, manifest neonatal hypotonia, feeding difficulties, hypogonadism, intellectual disability and sleep apnea^2^. However, individuals with SHFYNG have joint contractures, greater cognitive impairment, and a higher prevalence of autism spectrum disorder (ASD) than seen in PWS^3^. Additionally, SHFYNG is associated with a lower prevalence of hyperphagia and obesity than PWS^4^.

A hormonal phenotyping study of SHFYNG patients showed several similarities in biomarkers between SFHYNG and PWS, including low IGF1 and high ghrelin levels in patient serum, as well as alterations in glucose tolerance^5^. Some of these phenotypes, including low IGF1 and altered response to glucose tolerance tests, have been reported in mouse models of both SHFYNG and PWS as well^6–8^. Although it is still unclear which molecular alterations underlie the clinical phenotypes of SHFYNG and PWS, these studies suggest that the two disorders may share some causative molecular mechanisms, and exhibit a common theme of aberrations in growth factor response pathways.

The mammalian target of rapamycin (mTOR) is a serine/threonine kinase which forms two distinct complexes- mTORC1 and mTORC2, that mediate important cellular activities in response to various nutrients^9^. The mammalian target of rapamycin complex 1 (mTORC1) signaling pathway is a major regulator of cellular homeostasis downstream of growth factor and amino acid response. mTORC1 is involved in regulating many cellular functions including autophagy and lipid biogenesis, and is also known to play a role in neural dendrite formation^10,11^. Under normal conditions, growth factors, such as insulin, signal through protein kinase B (AKT) to increase mTORC1 activity^9^. This activity results in decreased autophagy, and increased lipid biogenesis. Conversely, a lack of growth factor signaling results in reduced activation of mTORC1, thus inducing autophagy, and inhibiting lipid biogenesis. The precisely controlled regulation of this pathway is necessary to maintain balanced cellular metabolism in response to environmental cues, and hyperactivation of mTORC1 signaling has been implicated in neurodevelopmental disorders such as autism and tuberous sclerosis complex (TSC, MIM 613254), as well as metabolic disorders such as obesity and type II diabetes^9,12^. Interestingly, both mTOR and the mTORC1 downstream target P-S6 were previously shown to be upregulated in a *Snord116del* PWS mouse model, while autophagy markers have been found to be downregulated in muscle tissue and POMC positive neurons of a Magel2 null mouse model^13,14^.

Although several studies have been published using patient-derived cell lines of individuals with PWS, there has been a lack of research carried out on SHFYNG patient-derived cell lines. Scarcity of human brain tissue samples from individuals with rare neurodevelopmental diseases, such as SHFYNG, necessitates the utilization of other primary cell models to perform molecular research on patient samples. One of the most accessible forms of human primary cells are fibroblasts, since they can be easily collected via skin biopsy. Fibroblasts themselves have proven to be a useful tool for investigation of neurological disease pathology^15^. However, fibroblasts can also be reprogrammed to induced pluripotent stem cells (iPSCs), which can then be differentiated into neurons (iNeurons) to better study neuron-specific disease phenotypes. iNeurons have been successfully used to model several neuropsychiatric disorders including PWS, idiopathic autism, and TSC^16–18^. As animal models of human neuropsychiatric disease have many known limitations, data from patient-derived primary cell lines are an important complement in identifying and understanding the pathological mechanisms of human diseases^19^. Studies in patient-derived primary cell lines, combined with those in animal models, can be used together to identify molecular pathways consistently altered between models. This not only increases the chances of experimental outcome reproducibility, but also allows for multiple modalities to test the efficacy of potential pharmacological treatments.

Here, we show that mTOR and downstream targets of mTORC1 are upregulated in the brains of Magel2 null mice, and in cultured fibroblasts of individuals harboring SHFYNG-causing mutations in *MAGEL2*. Additionally, both models show downstream impairments in autophagy and SHFYNG fibroblasts exhibit increased lipid droplet formation. Furthermore, neurons derived from SHFYNG patient iPSCs exhibit increased *mTOR* expression and altered dendrite formation. Importantly, these alterations are rescued by treatment with mTORC1 inhibitor rapamycin.

## Results

**Figure 1:**
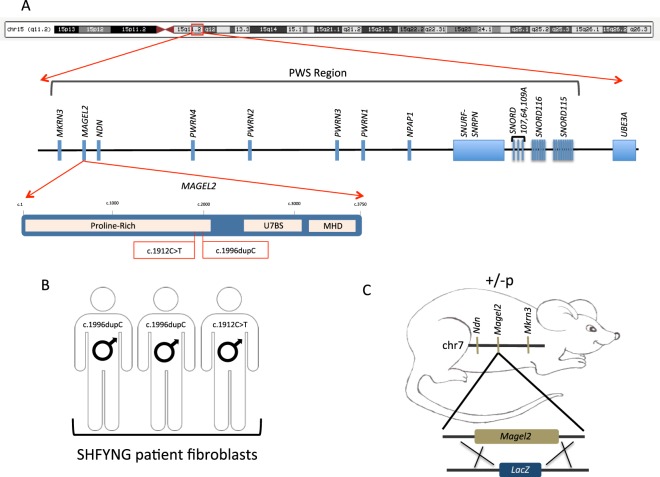
Human and mouse models of SHFYNG used in this study. (**A**) PWS region of human chromosome 15q11.2. MAGEL2 with each known major binding domain notated. SHFYNG causing nonsense or frameshift variants present in the patient cohort of this study are denoted with red lines. (**B**) Patient cell lines for this study were collected from three individuals. The disease-causing variant for each patient is noted. (**C**) The Magel2 null mouse model used in this study contains a LacZ knock-in, replacing Magel2 with LacZ under the endogenous Magel2 promoter. Mice inheriting the LacZ allele paternally (+/−p) are functional nulls and are referred to as Magel2 null.

SHFYNG patient cell lines used in this study were obtained from individuals diagnosed with SHFYNG due to clinical presentation and known truncating mutations in the transcriptionally active copy of *MAGEL2* (Fig. 1A,B). Details of patient cell lines can be found in the Materials and Methods section and in Supplemental Table 1.

### *116HG* is downregulated while mTOR is upregulated in *Magel2* null mouse hypothalamus

**Figure 2:**
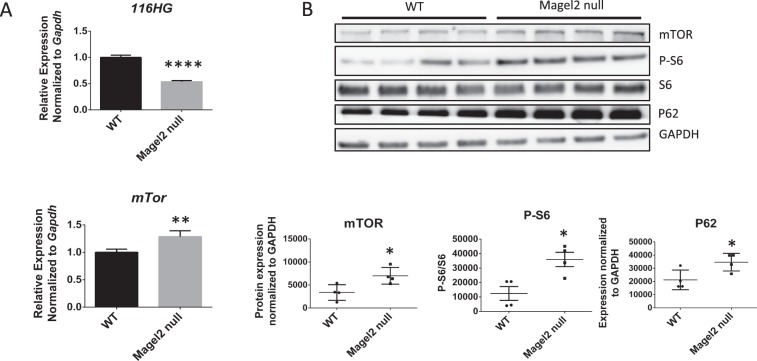
Increased expression of mTOR and downstream targets in Magel2 null mice. (**A**) *116HG* and *mTor* transcript levels in mouse hypothalamus. (**B**) Protein levels of mTOR, downstream signaling target P-S6 and autophagy marker P62 in mouse hypothalamus. Data are represented as the mean ± SEM. T-tests were performed on all data sets. *p < 0.05, **p < 0.01, ****p < 0.0001. For qRT-PCR and western blot data from mouse hypothalamus – WT: n = 4, Magel2 null: n = 4. Data is representative of 2–4 experiments.

*116HG*, the long non-coding RNA originating from the same portion of the PWS region as the SNORD116 snoRNAs, has previously been shown to interact with and negatively regulate transcription of *mTor*, with a *SNORD116del* PWS mouse model exhibiting increased *mTor* expression^13^. We found that *116HG* expression is downregulated by ~50%, while *mTor* expression is upregulated in *Magel2* null mouse hypothalamus (Fig. 2A). Accordingly, *Magel2* null mouse hypothalamus showed increased levels of mTOR protein (Fig. 2B). Our results also showed elevated levels of phospho S6, a downstream substrate of mTORC1^20^, suggesting an increase in mTORC1 activity in Magel2 null mouse hypothalamus (Fig. 2B). Another downstream substrate of mTORC1, P-4EBP1, is also upregulated in Magel2 null mouse hypothalamus (Supplementary Fig. 1), further supporting the hypothesis that mTORC1 activity is increased in this model. P62 is commonly used as a readout of autopghagy^21^, and has previously been shown to be upregulated in *Magel2* null mouse muscle tissue and POMC positive neurons^14^. Consistent with these studies, we also found that P62 is increased in *Magel2* null mouse hypothalamus, indicating an impairment of autophagy in this model (Fig. 2B).

### mTORC1 activity is high and autophagy is decreased in SHFYNG patient fibroblasts

**Figure 3:**
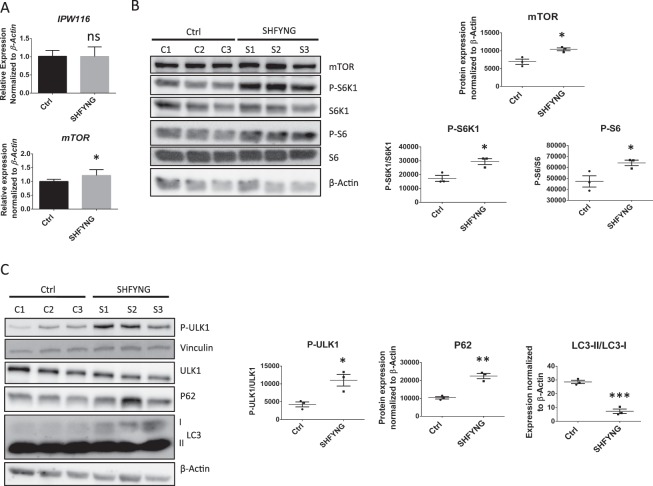
Increased mTORC1 activity and altered expression of autophagy markers altered in SHYNG patients’ fibroblasts. (**A**) *IPW116* and *mTOR* transcript levels in human fibroblasts. (**B**) Protein levels of mTOR and downstream signaling targets in human fibroblast lines. (**C**) Protein levels of autophagy markers downstream of mTORC1 signaling in human fibroblasts. Data are represented as the mean ± SEM. T-tests were performed on all data sets. *p < 0.05, **p < 0.01, ***p < 0.001. For qRT-PCR and western blot data from human fibroblasts – Ctrl: n = 3 individuals, SHFYNG: n = 3 individuals. Data is representative of 2–4 experiments.

We found that *mTOR* expression was significantly increased in SHFYNG fibroblasts, while expression of *IPW116*, the human homolog of *116HG*, was unchanged (Fig. 3A). SHFYNG patient fibroblasts also showed increased levels of mTOR protein as well as increased phosphorylation levels of the mTORC1 substrates S6K1, S6, and 4EBP1 (Fig. 3B and Supplementary Fig. 2). It has been shown that P-AMPK is a negative regulator of mTORC1 activity and a positive regulator of autophagy^22^. Therefore, we assessed P-AMPK levels in this model and found a significant decrease of P-AMPK levels in SHFYNG fibroblasts compared to controls (Supplementary Fig. 2). This decrease in P-AMPK may contribute to increased mTORC1 activity independently of mTOR protein levels, and to reduction of autophagy independently of mTORC1 activity in SHFYNG patient fibroblasts.

It is well known that autophagy is negatively regulated by mTORC1^23^, and that this regulation occurs partially through phosphorylation and inhibition of ULK1^10^. Active ULK1 is involved in autophagosome formation, and phosphorylation by mTORC1 inhibits this function^24^. We show that phosphorylation of ULK1 is upregulated in SHFYNG patient fibroblasts (Fig. 3C). SQSTM1/P62 binds and delivers autophagy substrates to the lysosome for degradation, and is itself constitutively degraded by the lysosome, making its abundance a widely used readout of autophagic flux^25^. Like *Magel2* null mouse hypothalamus, SHFYNG patient fibroblasts exhibited increased levels of P62, indicating decreased autophagic function (Fig. 3C). The conversion of LC3-I to LC3-II is critical in autophagosome formation, with LC3-II playing an active role in maturation of the autophagosome^26^. We show that LC3-II/LC3-I ratio is significantly decreased in SHFYNG patient fibroblasts (Fig. 3C), suggesting a decrease in autophagosome formation in SHFYNG patient fibroblasts.

**Figure 4:**
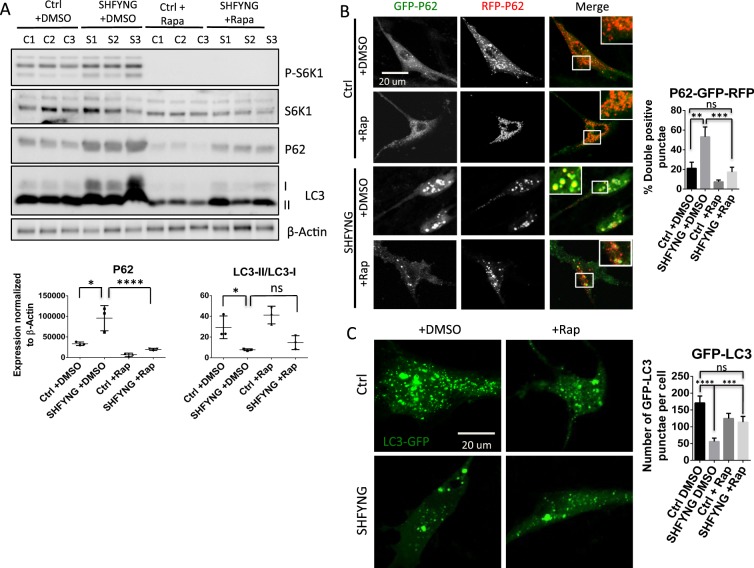
Decrease of autophagic flux in SHFYNG patient fibroblasts is rescued by treatment with rapamycin. (**A**) Protein levels of autophagy markers in human fibroblasts treated with DMSO or 300 nM rapamycin for 24 hrs. (**B**) Human fibroblasts transfected with P62-GFP-RFP expressing plasmid and treated with DMSO or 300 nM rapamycin for 24 hrs. GFP fluorescence is quenched by low lysosomal pH upon uptake into the lysosome. GFP-RFP double positive punctae, seen as yellow, show accumulation of P62 not being digested by the lysosomes. Percent of GFP-RFP double positive punctae in relation to total punctae is quantified. Representative images from Ctrl and SHFYNG groups treated with either DMSO or rapamycin are shown. (**C**) Human fibroblasts transfected with LC3-GFP expressing plasmid treated with DMSO or 300 nM rapamycin for 24 hrs. Accumulation of LC3 indicates induction of autophagy. Total number of LC3-GFP punctae per cell is quantified. Representative images from Ctrl and SHFYNG groups treated with either DMSO or rapamycin are shown. Data are represented as the mean ± SEM. One-way ANOVA corrected for multiple comparisons was performed on all data sets. *p < 0.05, **p < 0.01, ***p < 0.001, ****p < 0.0001. For western blot and immunofluorescence data from human fibroblasts – Ctrl: n = 3 individuals, SHFYNG: n = 3 individuals. Bar diagrams reperesent quantification of Ctrl: n = 3 individuals, SHFYNG: n = 3 individuals. Data is representative of 2–4 experiments.

Rapamycin is a specific inhibitor of mTORC1 and is widely used to inhibit mTORC1 activity^27^. Treatment with rapamycin has previously been shown to inhibit phosphorylation of mTORC1 substrate, S6K1, and induce autophagy in cell culture models^28^. 24 hrs treatment with 300 nM rapamycin eliminated phosphorylation of S6K1 and restored markers of autophagy in SHFYNG patient fibroblasts to similar levels as those seen in control fibroblasts (Fig. 4A). P62 levels were decreased, while LC3-II/LC3-I ratio was significantly increased in SHFYNG fibroblasts treated with rapamycin (Fig. 4A). P62-GFP-RFP construct was used to monitor P62 uptake into the lysosome, which is reflective of the rate of autophagic flux. When the P62-GFP-RFP protein is taken up by the lysosome, the GFP is quenched by the low lysosomal pH, while the RFP is stable and visible in the lysosomal compartment^29^. SHFYNG fibroblasts transiently transfected with the P62-GFP-RFP construct exhibited a significant increase in the levels of double positive (GFP-RFP positive) puncta compared to transfected control fibroblasts (Fig. 4B). This increase in double positive puncta was reduced to control levels upon rapamycin treatment (Fig. 4B). These data support the observation that P62 is not being efficiently degraded by the lysosome in SHFYNG fibroblasts, but that inhibition of mTORC1 by rapamycin treatment restores autophagy to normal levels. Treatment of the fibroblasts with the potent autophagic flux inhibitor, bafilomycin^21^, was performed as a control and shows that inhibition of autophagy results in a very high percentage of GFP-RFP double positive punctae (Supplementary Fig. 3). Importantly, rapamycin-mediated decrease in the number of GFP-RFP double positive punctae was completely inhibited by post-treatment with bafilomycin in SHFYNG patient fibroblasts, confirming the defects in autophagic flux in SHFYNG patients (Supplementary Fig. 3). GFP-tagged LC3 (GFP-LC3), when transfected into a cell line, is also used as a readout of autophagosome formation, with increased GFP-LC3 puncta indicating increased autophagosome formation and vice-versa^30^. SHFYNG fibroblasts transfected with the GFP-LC3 construct showed a significant decrease in the number of GFP positive punctae compared to control fibroblasts, further supporting a pattern of reduced autophagy in SHFYNG fibroblasts (Fig. 4C). Rapamycin treatment showed a significant rescue in the number of GFP-LC3-positive punctae in SHFYNG fibroblasts (Fig. 4C). Together, these results indicate that both *Magel2* null mice and SHFYNG patient fibroblasts exhibit impaired autophagy, and that this impairment can be rescued by inhibition of mTORC1 activity.

### SHFYNG patient fibroblasts exhibit increased lipid droplet formation

**Figure 5:**
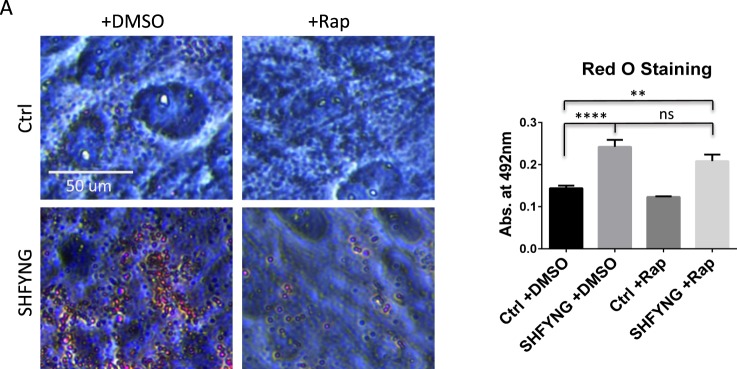
SHFYNG patient fibroblasts exhibit increased lipid droplet accumulation. (**A**) Representative images of Oil Red O staining of lipid droplets in human fibroblasts treated with DMSO or 300 nM rapamycin for 24 hrs. Data are represented as the mean ± SEM. One-way ANOVA corrected for multiple comparisons was performed on this data set. **p < 0.01, ****p < 0.0001. Ctrl: n = 3 individuals, SHFYNG: n = 3 individuals. Data is representative of 2 experiments.

mTOR is also known to be a positive regulator of lipid synthesis^9^. Due to the fact that that models of SHYFNG exhibit increased fat mass and decreased lean mass, we investigated lipid synthesis in SHFYNG patient fibroblasts^5,31^. Oil Red O staining was used to assess lipid droplet formation in fibroblast cultures^32^. SHFYNG fibroblasts exhibited a greater number of lipid droplets compared to controls (Fig. 5). Quantification by lysing the lipid droplets and measuring the intensity of the dye released from each culture confirmed that SHFYNG fibroblasts had taken up more Oil Red O stain than controls (Fig. 5). This increase was partially, but not significantly, reduced upon rapamycin treatment indicating that mTORC1 may play a role in SHFYNG related lipid imbalance.

### SHFYNG iNeurons exhibit increased *mTOR* expression and decreased dendrite formation

**Figure 6:**
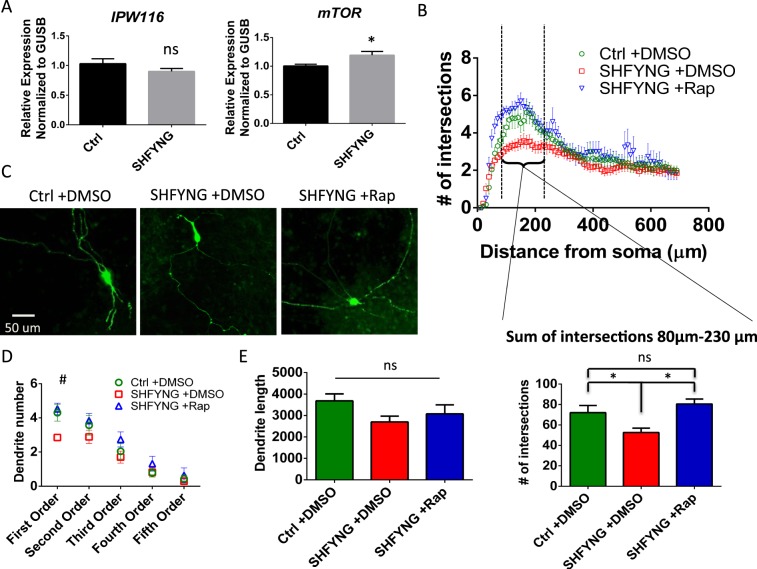
Abnormal dendritic arborization of SHFYNG patient iPSC derived neurons is rescued by rapamycin treatment. (**A**) IPW116 and mTOR transcript levels in human iPSC derived neurons (iNeurons). (**B**) Sholl analysis of human iNeurons - average number of dendritic intersections per concentric circle every 10 µm is plotted. The graph below the Sholl plot represents the average sum of dendritic intersections with shells between 80 µm and 230 µm. (**C**) Representative images of NGN2-GFP infected iNeurons used for neuronal tracing. (**D**) Average number of dendrites per iNeuron plotted by branching order. (**E**) Average total dendritic length of iNeurons. Where indicated, iNeurons were treated with DMSO or 100 nM rapamycin for 84 hrs. Data are represented as the mean ± SEM. T-test was performed on data sets in panel A. One-way ANOVA corrected for multiple comparisons was performed on data sets in panels B, D, and E. *p < 0.05, ^#^p < 0.05 between Ctrl + DMSO and SHFYNG + DMSO. For panel A, Ctrl: n = 4 (2 individuals × 2 clones each), SHFYNG: n = 4 (2 individuals × 2 clones each). For panels B-E, Ctrl: n = 4 (2 individuals × 2 clones each), SHFYNG: n = 3 (2 individuals – 1 individual × 2 clones; 1 individual × 1 clone). Data is a combination of 2 experiments.

mTOR is known to play an important role in neuronal growth and development, and previous studies have shown that increased activation of mTORC1 affects dendritic formation and branching^11^. qRT-PCR analysis of D30 iNeurons showed increased expression of *mTOR* with no change in expression of *IPW116* in SHFYNG iNeurons compared to controls (Fig. 6A). This set of data closely matches what is seen in SHFYNG fibroblasts (Fig. 3A). Sholl analysis^33^ revealed that SHFYNG iNeurons have fewer dendritic intersections 80 µm–230 µm from the soma (Fig. 6B,C), and a decreased number of first order dendrites compared to controls (Fig. 6D). The sum of the dendritic intersections between 80 µm–230 µm was quantified and plotted, as this is the region displaying the most difference in dendritic complexity between SHFYNG and control lines. These changes are rescued by 84 hrs of 100 nM rapamycin treatment (Fig. 6B–D). SHFYNG iNeurons showed a partial, but statistically insignificant decrease in dendrite length (Fig. 6E).

**Figure 7:**
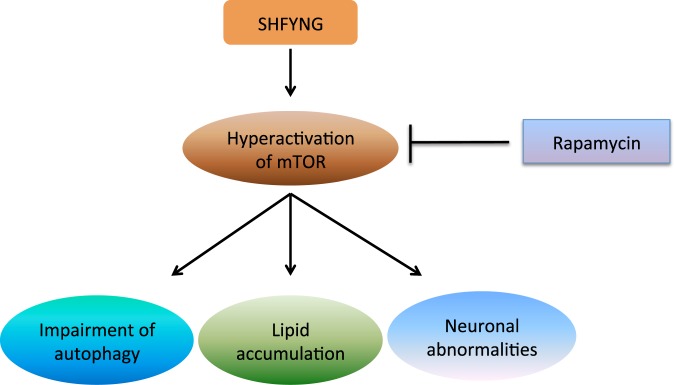
Schematic of pathogenic abnormalities in SHFYNG patients.

Cumulatively, our findings suggest that SHFYNG patient cell lines exhibit increased mTOR expression and mTORC1 activity, as well as corresponding downstream alterations in autophagy, lipid droplet formation and neuronal morphology (see model in Fig. 7). These alterations are fully or partially rescued upon inhibition of mTORC1 activity by treatment with rapamycin. Importantly, increased levels of mTOR and mTORC1 targets, as well as alterations in autophagy markers are also seen in Magel2 null mice, indicating that some molecular phenotypes of Magel2 null mice recapitulate those seen in SHFYNG patients.

## Discussion

In this study, we identified an upregulation of mTOR and its downstream targets, as well as alteration of the autophagy marker P62 in *Magel2* null mouse hypothalamus. We recapitulated and expanded upon these findings in human SHFYNG patient fibroblasts. We also found that lipid droplet formation, which is positively regulated by mTORC1 signaling, is increased in SHFYNG patient fibroblasts. Additionally, we observed altered dendrite formation in iNeurons from SHFYNG patients. Importantly, we showed that all identified phenotypes downstream of mTORC1 signaling could be fully or partially rescued by rapamycin treatment of the patient derived cell lines.

Identification of the molecular alterations underlying the phenotype of SHFYNG has been challenging for a number of reasons. Although there are two *Magel2* null mouse models on which many studies have been published, it is unclear how findings in these models translate to the human phenotype. This is partially due to the fact that it is unknown if any truncated MAGEL2 protein product is produced in SHFYNG patients, and if yes whether that truncated protein has any function. The gene mutations that cause SHFYNG are nonsense or frameshifting mutations in *MAGEL2*, and they result in the presence of a premature stop codon. However, because *MAGEL2* is a single exon gene, it is unlikely that the truncated product is undergoing nonsense mediated decay. Nevertheless, it is still quite possible that no functional protein is produced. Even if there is any product, it may be rapidly degraded due to aberrant folding. Unfortunately, absence of a reliable MAGEL2 antibody limits the possibility to test this hypothesis in patient cell lines. It has previously been shown that *MAGEL2* is expressed in human fibroblasts, and the relative ease of skin biopsies compared to other tissue collection makes these cells a good choice for molecular studies on human samples^34^. Here, we show that mTOR and its downstream effectors are altered in both Magel2 null mice and SHFYNG patient fibroblasts in a manner similar to that previously seen in a PWS mouse model^13^. This suggests that at least some molecular changes in SHFYNG patient cell lines correspond to those seen in a Magel2 null model.

mTOR is a serine/threonine kinase and acts downstream of growth factor signaling to modulate cellular homeostasis^9^. It has previously been shown that response to and levels of growth factors are altered in both human patients and mouse models of PWS and SHYFNG^5–8^. These include alterations in serum ghrelin and IGF1 levels as well as abnormal response to glucose tolerance testing, suggesting that growth factor signaling pathways are likely disrupted in these disorders. A previous study showed that the expression level of mTOR and phosphorylation levels of its downstream target S6 are upregulated in a *Snord116del* mouse model of PWS^13^. This study also showed that *116HG*, the long non-coding transcript originating from the *Snord116* region, interacts with *mTor* and competitively binds the transcriptional activator RBBP5. Upon loss of *Snord116*, this competitive binding is lost and *mTor* is transcriptionally upregulated. In humans, this long non-coding transcript is referred to as *IPW116*, and behaves similarly to *116HG* in regards to its impact on *mTOR* expression. In our study, we show transcriptional downregulation of *116HG* in *Magel2* null mouse hypothalamus, and a concurrent increase in *mTor* expression. Interestingly, in SHFYNG patient fibroblasts and iNeurons, *mTOR* expression is upregulated, though there is no significant difference in *IPW116* expression levels. It is possible that loss of MAGEL2 in human cells leads to alterations in chromatin looping and 3D conformation of genomic architecture in this region, and that this could affect interaction of *IPW116* with *mTOR*, resulting in transcriptional upregulation of *mTOR* without affecting transcript levels of *IPW116*. It is also possible that the upregulation of *mTOR* transcript in both human and mouse tissues is the result of a mechanism independent of 116HG/IPW116 function. Although we do see a ~50% downregulation of 116HG transcript in *Magel2*-null mice, we do not know if this decrease in expression is responsible for driving the increase in mTOR transcript. Further studies addressing these questions should be performed in the future. Additionally, the increase in *mTOR* transcript is small compared to the downstream effects on mTORC1 signaling. This discrepancy could be the result of alterations in factors impacting activity levels of mTORC1 independently of mTOR protein levels. For instance, TSC is a disorder resulting from deleterious mutations in the *TSC1/2* genes, which results in hyperactivation of mTORC1 signaling^21^. This hyperactivation occurs in absence of increased *mTOR* transcript or protein. In addition to signaling pathways that control mTORC1 activity through regulation of TSC1/2, there are also pathways such as the amino acid signaling pathway and P-AMPK, which can regulate mTORC1 activity independently of AKT activity and mTOR levels^30,35^. In this study we show that P-AMPK levels are decreased in fibroblasts for SHFYNG patients. Therefore, hyperactivity of mTORC1 in these cells could be contributed to, at least in part, by decreased negative regulation by P-AMPK. Additionally, the alterations in autophagy observed in this study could have contributions independent of mTORC1 signaling. For example, it is known that MAGEL2 is involved in regulation of retrograde transport of CI-M6PR, which plays an important role in the delivery of acid hydrolases to lysosomes^36,37^. Thus, altered trafficking of M6PR may contribute to impaired autophagy through dysregulated transport of acid hydrolases to the lysosome. Future studies in this area should explore upstream mechanisms leading to mTORC1 hyperactivation and mTORC1 independent mechanisms of impaired autophagy in SHFYNG.

mTORC1 is a critical regulator of autophagy, and its activation is known to suppress autophagy through several mechanisms^10^. Precise control of autophagic flux is necessary for maintenance of cellular balance between protein production and degradation, and alterations in autophagy have been implicated in many disorders, including metabolic and neurodevelopmental diseases^38^. It has previously been shown that Magel2 null mice exhibit increased P62 levels in skeletal muscle and POMC positive neurons, indicating impaired autophagy in this model^14^. Our study confirms this finding by showing increased levels of P62 in Magel2 null mouse hypothalamus. Additionally, we have shown that autophagic function is reduced in SHFYNG patient fibroblasts and that rapamycin treatment of patient fibroblast lines inhibits mTORC1 activity and rescues the autophagy phenotype partially or completely in all assays. This indicates that hyperactivation of the mTORC1 pathway is likely a major contributor to the reduction in autophagy seen in SHFYNG patient fibroblasts, and that pharmacological inhibition of mTORC1 can restore autophagy to normal levels.

It has been shown that mTORC1 and autophagy play a role in both fat and muscle development and maintenance. Infants with both SHFYNG and PWS exhibit severe hypotonia, and human patients as well as animal models of these disorders exhibit high fat mass to lean mass ratio in adolescence and adulthood^5,31^. Our results show that SHFYNG patient fibroblasts exhibit increased presence of lipid droplets compared to controls. Inability of rapamycin to rescue this phenotype may be due to the short (24 hr) treatment period. As clearance of excess lipid droplets likely takes greater than 24 hrs, a longer treatment period may be necessary to reduce lipid droplets to control levels. It is also important to recognize that pathways not immediately downstream of mTORC1 activity may contribute to the lipid accumulation seen in the SHFYNG patient fibroblasts. For example, it is known that AMPK promotes lipid and sterol synthesis in the cell^35^. In this study we show that P-AMPK levels are reduced in SHFYNG fibroblasts, thus implicating the AMPK pathway as a potential contributor to increased lipid accumulation in these cells. Additionally, it has been shown that autophagy plays a role in degrading intracellular lipid stores, and that loss of autophagy leads to increased cellular lipid accumulation^39^. Therefore, the impairment of autophagy observed in the SHFYNG cell lines may also contribute to lipid accumulation, as lipids may not be effectively cleared and recycled in cells with deficits in autophagy.

Increased activity of mTORC1 has been linked to neuropsychiatric disorders including TSC, Fragile X Syndrome (FXS, MIM 300624), PTEN-related ASD and intellectual disability (ID), and idiopathic autism^9,12,40,41^. In addition to general developmental delay and ID, SHFYNG patients exhibit a high prevalence of autism, with 78% receiving an ASD diagnosis, and a low average IQ (Avg IQ = 38)^3^. While the alterations in bone mineral density and fat mass to lean mass are similar between SHFYNG and PWS, the neuropsychiatric and developmental phenotypes associated with SHFYNG are more severe than in PWS. While PWS is typically associated with mild ID, the study by McCarthy *et al*. (2019) reported an average IQ of 38 in a cohort of SHFYNG patients, with 25% of these individuals having IQ scores less than 20^3^. This is quite low relative to PWS, which has a reported average IQ of 60–70. Importantly, IQ scores below 70 indicate potential ID with scores below 20 indicating profound disability, highlighting the degree of cognitive impairment associated with SHFYNG^42^. SHFYNG individuals also meet developmental milestones later, taking longer to sit, crawl, walk, and talk compared to individuals with PWS^3^. The results of these studies show that while there are many similarities between PWS and SHFYNG, there are also distinct phenotypic differences, indicating that the pathomechanisms underlying these diseases may overlap but are likely not identical.

To assess neuron-specific phenotypes of SHFYNG, we used an established direct conversion protocol to transdifferentiate SHFYNG patient iPSCs into iNeurons. This protocol uses viral overexpression of NGN2 to produce a pure neuronal culture of primarily excitatory neurons^43,44^. Neuronal dendrites receive sensory and synaptic input from their surrounding environment and neighboring neurons, and the dendritic branching pattern impacts the inputs received by a neuron^45^. Thus, changes in dendritic morphology can affect information processing and neuronal activity. Our results show that SHFYNG iNeurons exhibit increased expression of *mTOR*, and decreased dendritic formation, specifically affecting dendrites originating from the soma (first order dendrites). These findings align with those seen in some other genetic disease models resulting in hyperactivation of mTORC1. Specifically, loss of SIRT1 results in increased mTORC1 activity, and primary neuronal cultures from a Sirt1 null mouse exhibit decreased dendritic formation^11^. This altered dendritic formation can be rescued by rapamycin treatment in the Sirt1 null model, similarly to what we see in rapamycin treated SHFYNG neurons. Interestingly, a previous study showed that the same *Magel2*-null mouse model used here displays decreased frequency of excitatory post-synaptic currents and decreased AMPAR mediated currents in hypothalamic oxytocin neurons^46^. The authors of this study did not perform a morphological Sholl analysis on these neurons, but studies in other models of neurodevelopmental disorders suggest there may be a positive correlation between dendritic complexity and current frequency in neuronal signaling^47^. Additionally, this *Magel2*-null mouse model exhibits decreased excitatory leptin signaling in POMC positive neurons, while inhibitory leptin signaling remained unchanged^48^. These reports suggest the need for future investigations focused on excitatory synaptic function in *Magel2*-null models. Similarly, future studies on SHFYNG patient derived neurons should investigate the presence of neurophysiological and synaptic phenotypes, and the potential of rapamycin to treat any abnormalities observed.

In conclusion, we show increased levels of mTOR and its downstream readouts of activity, as well as altered autophagy in animal and human models of SHFYNG. Additionally, we show deficits in dendrite outgrowth in SHYFNG patient iNeurons. Future studies investigating the mechanistic role of MAGEL2 in mTORC1 hyperactivation are necessary. However, given that short-term rapamycin treatment rescues the phenotypes reported here, future studies investigating the therapeutic potential of rapamycin and its FDA approved analogues in animal models of SHFYNG and PWS are warranted.

## Methods

### Research guidelines and regulations

All research and animal care procedures were approved by the Baylor College of Medicine Animal Care and Use Committee and were performed in accordance with the relevant guidelines and regulations (protocol #AN-6922).

Studies recruiting human subjects were approved by the Institutional Review Board at Baylor College of Medicine, and subjects or subjects’ parents and/or legal guardians consented to use of their cells in research. Informed consent for study participation was obtained from all patients. All patients have been consented under human research protocol H-34578 under the Baylor College of Medicine IRB. All research was performed in accordance with relevant guidelines and regulations.

### Animals

Mice were maintained on a 14 hrs light/10 hrs dark cycle with *ad libitum* access to water and regular mouse chow. Magel2 null mice and wildtype (WT control) littermates (C57BL/6-Magel2tm1Stw/J, The Jackson Laboratory stock #009062) greater than 6 months of age at time of tissue harvest were used for experiments. Mice carrying a paternally inherited Lac-Z knock-in allele (*Magel2*+/−*p*) are functionally null for Magel2 and are referred to here as Magel2 null (Fig. 1C). Genotyping was carried out as previously described^49^. All research and animal care procedures were approved by the Baylor College of Medicine Animal Care and Use Committee and were performed in accordance with the relevant guidelines and regulations (protocol #AN-6922).

Whole mouse hypothalamus was harvested, snap frozen in a dry ice/ethanol bath, and stored at −80C until protein or RNA extraction.

### Human subjects

Human fibroblast lines were obtained from skin biopsies of SHFYNG patient and control individuals. Studies recruiting human subjects were approved by the Institutional Review Board at Baylor College of Medicine, and subjects consented to use of their cells in research. Informed consent for study participation was obtained from all patients. All patients have been consented under human research protocol H-34578 under the Baylor College of Medicine IRB. Patients were recruited based upon presence of nonsense or frameshift mutations in *MAGEL2*, identified by whole exome sequencing. Inheritance pattern was verified either by sequencing of the patient’s father or by the SmaI restriction digest and sequencing method described in Schaaf *et al*.^1^.

For experiments on fibroblast lines: A total of three patients and three WT (controls) were used for all experiments performed on human fibroblasts. SHFYNG 1 and SHFYNG 2 each carry a frameshifting 1196dupC variant in the transcriptionally active copy of *MAGEL2* (Fig. 1A,B and Supp. Table 1). SHFYNG 1 has been previously reported as Patient 1 in Fountain *et al*. (2016) and Patient 8 in McCarthy *et al*. (2018), while SHFYNG 2 was previously reported in Fountain *et al*. (2016) as Patient 12^4,5^. SHFYNG 3 carries a nonsense 1912C > T variant in the transcriptionally active copy of *MAGEL2* (Fig. 1A,B), and was previously reported in Schaaf *et al*. (2013) as Patient 2^1^. Control 1 and Control 3 are unrelated, unaffected siblings of individuals with other genetically inherited neurodevelopmental disorders. Control 2 is a commercially available control fibroblast line from Coriell repositories. See Supplemental Table 1 for human subjects details.

For experiments on iPSC derived neurons: A total of two patients (two clones from one individual and one clone from the second individual) and two controls (two clones from each individual) were used for all experiments performed on iPSC-derived neurons. SHFYNG 1 and SHFYNG 2 are derived from the same individuals as fibroblast lines SHFYNG 1 and SHFYNG 2. Control 3 was derived from the same individual as fibroblast line Control 3. Control 4 was received from the Baylor College of Medicine Stem Cell Core. See Supplemental Table 1 for human subjects details.

### Generation of human induced pluripotent stem cells (iPSCs)

SHFYNG 1–2, and Ctrl 3 fibroblast lines were derived from subject skin biopsies by the IDDRC Tissue Culture Facility Core. Fibroblasts were reprogrammed into iPSCs by the Baylor College of Medicine Human Stem Cell Core (HSCC) using the CytoTune-iPS 2.0 Sendai Reprogramming Kit (Life Technologies), following the manufacturer’s protocol. Following transduction, cells were grown in high-glucose DMEM supplemented with 10% fetal bovine serum and 1 × MEM non-essential amino acids (Life Technologies) for 5 days, in TeSR-E7 (StemCell) for 8 days, and in TeSR-E8 (StemCell) until day 21 when pluripotent colonies were manually picked. Lower passage-number colonies were picked manually, while later passages were performed using ReLeSR (StemCell) or 0.5 mM EDTA. After reprogramming, iPSCs were maintained in mTeSR1 (StemCell Technologies) on Matrigel (354277, Corning) or Cultrex (3433-010-01, Trevigen). All cells were incubated at 37 °C with 95% humidity and 5% CO_2_.

The Control 4 iPSC line was previously reprogrammed by the HSCC and referred to as HSCC-003iPS in Liu *et al*.^50^.

Immunofluorescence and qRT-PCR for pluripotency markers were performed on all iPSC lines; representative data is shown in Supplemental Fig. 4A,C. Additionally, flow cytometry for pluripotency markers SSEA4 and OCT4 were performed on each iPSC line (data not shown). Each iPSC line was shown to have a normal karyotype (data not shown).

### Vector design

To make the human NGN-2 inducible expression plasmids used in this study, we modified pLV-TetO-hNGN2-eGFP-Puro plasmid (Addgene # 79823), which was a gift from Kristen Brennand and was described before by Ho *et al*.^44^. Briefly, we used restriction cloning to first replace the T2A-puromycin cassette between the BsrGI and NheI sites with a DNA sequence for T2A-blasticidin (custom gene synthesis from Integrated DNA Technologies). We named the resulting plasmid pLV-TetO-hNGN2-eGFP-BSD or just hNGN2-eGFP in this manuscript. Next, from this new plasmid we removed the EGFP portion between the XbaI and BsrGI restriction sites and replaced it with accordingly digested mCherry fragment that was PCR amplified from pAAV-hSynI-mCherry-WPRE-hGH poly(A) plasmid (Addgene #71650). This second vector was named pLV-TetO-hNGN2-mCherry-BSD or shortly hNGN2-mCherry.

### Lentivirus production

We transfected HEK-293FT cells at 70–80% confluence using jetPRIME (114–15, Polyplus-transfection) and following transfection reagent protocol. Together with each target plasmid, we used the second-generation lentiviral packaging plasmids (gift from Dr. Didier Trono.) consisting of the two helper plasmids psPAX2 with minimal HIV enzyme set (Addgene #12260) and pMD2.g coding for VSV-G envelope (Addgene #12259). The lentivirus-containing supernatant was collected at 24- and 48-hour points and concentrated overnight at 4 °C according to Lenti-X Concentrator (631232, Takara) protocol. In order to determine the appropriate viral titer, single cell suspension of 2 × 10^6^ H9 ESCs in mTeSR1 medium with 10 µM Y-27632 (S1049, Selleck Chemicals) and 6 µg/ml Polybrene (H9268, Millipore Sigma) were co-infected with various amounts of FUdeltaGW-rtTA^51^ (gift from Konrad Hochedlinger, Addgene #19780) and hNGN2-eGFP or hNGN2-mCherry virus. These cell suspensions were then incubated overnight each in a single well of a low-binding 6-well plate (657970, Greiner Bio-One) with 70 rpm set on an orbital shaker, then moved to matrigel-coated plates and after attachment induced with 2 µg/ml of doxycycline (D9891, Millipore Sigma). The optimal titer was determined on the following day based on the infection efficiency. We selected titer combinations which resulted in lower cytotoxicity and 85–95% fluorescently labeled ESC which theoretically corresponds to MOI of 2–3.

### Generation of iNeurons

iPCSs were differentiated into neurons following a modified version of the direct differentiation protocol described by Zhang *et al*., Thoma *et al*. and Ho *et al*.^43,44,52^. Briefly, 2 × 10^6^ iPSCs in mTeSR1 with 10 µM Y-27632 and 6 µg/ml Polybrene were infected with two lentiviral constructs, pLV-TetO-hNGN-2-mCherry-BSD and FUdeltaGW-rtTA using the established viral titers. The iPSCs were incubated in suspension on a shaker overnight and were moved to matrigel-coated plates the following day. The iPSCs were then expanded for several days in mTeSR1 medium, passaged with Accutase (A6964, Millipore Sigma), counted, and plated at a density of 0.125 × 10^6^ cells/cm^2^ in 12-well tissue culture plates and 8-well glass chamber slides (PEZGS0816, Millipore Sigma). The iPSCs were induced on the following day (Day 0) with Neural Induction Medium (NIM – DMEM/F-12:Neurobasal (1:1) with 1X Penicillin-Streptomycin, 2% B-27 without vitamin A, 1% N-2, 2 mM Glutamax, all from ThermoFisher) supplemented with doxycycline (2 µg/ml), and SMAD inhibitors, 1 µM dorsomorphin (sc-361173, Santa Cruz Biotechnology) and 10 µM SB431542 (S1067, Selleck Chemicals). On Day 2, the culturing medium was changed to Neural Differentiation Medium (NDM - Neurobasal medium with 1X Penicillin-Streptomycin, 2% B-27 without vitamin A, 2 mM Glutamax, - all from ThermoFisher, 20 ng/ml BDNF, 10 ng/ml GDNF, 10 ng/ml NT-3 (all three were human animal free peptides from Peprotech), 100 μM db-cAMP (sc-201567B, Santa Cruz Biotechnology), and 200 μM ascorbic acid (A8960, Millipore Sigma) supplemented with doxycycline, and 20 µg/ml of blasticidin (ant-bl-05, Invivogen) for selection. Blasticidin was kept in the medium from Day 2 to Day 8. The cultures were supplemented with 1 µg/ml laminin (3400-010-02, Travigen) once every 4 days and with matrigel once every 8 days to prevent detachment. For each line, one glass chamber slide was re-infected with pLV-TetO-hNGN-2-EGFP-BSD at a 500-fold dilution of normal viral titer to sparsely label neurons with GFP for tracing and analysis. Following differentiation, the culture medium was changed every 2–3 days. The cells were kept in culture for a total of 30 days.

iNeurons were treated with DMSO or 100 nM rapamycin for 84 hrs before harvest. Fresh DMSO or rapamycin was added with each media change.

Immunoflourescence for neuronal marker β-III-tubulin, and qRT-PCR for neuronal markers *VGLUT1* and *NCAM* were performed on all D30 iNeurons; representative data is shown in Supplemental Fig. 4B,C.

### Cell culture

Human fibroblasts were cultured in high-glucose DMEM (Thermo Fisher Scientific) supplemented with 20% heat inactivated fetal bovine serum (Atlanta Biological). Media was changed every 2–3 days. For experiments with a rapamycin treated group, cells were grown to 90% confluency and treated with either DMSO (Sigma) or 300 nm Rapamycin for 24 hrs prior to harvest. Cells were incubated at 37 °C with 95% humidity and 5% CO_2_.

### Plasmids and fibroblast transfection

P62-GFP-RFP and LC3-GFP plasmids were a generous gift from Dr. Marco Sardiello and have been previously used in publications from his lab^29,30^. Transfection of human fibroblasts was performed with Lipofectamine 2000 (Thermo Fisher Scientific) or jetPRIME (Polyplus) transfection reagent. Transfection was carried out according to manufacturer’s protocol. Cells were treated with DMSO (Sigma) or 300 nM rapamycin 24 hrs post transfection, and harvested 48 hrs post transfection.

### RNA isolation and qRT-PCR

RNA isolation was performed using the miRNeasy mini kit (QIAGEN) according to manufacturer’s instructions. cDNA was synthesized using M-MLV Reverse Transcriptase (Invitrogen) according to the manufacturer’s directions. qRT-PCR reactions were run in triplicate using SYBER Green master mix (BioRad) according to manufacturer’s protocol. qRT-PCR was performed using the CFX96 Real Time System (Bio-Rad). qRT-PCR primer sequences are provided in Supplemental Table 2.

### Immunoblotting

Human fibroblasts were rinsed once with cold PBS and harvested with NP-40 lysis buffer, supplemented with 1X protease and phosphatase inhibitors (Thermo Fisher Scientific). Mouse hypothalamus was lysed in NP-40 lysis buffer supplemented with 1X protease and phosphatase inhibitors (Thermo Fisher Scientific) and pulverized using a handheld pellet pestle tissue homogenizer (Sigma). Protein concentration was measured using a BCA protein assay kit (Pierce) or Bradford reagent (Sigma) using BSA as a standard for both methods. Lysates were separated on SDS-PAGE gels and transferred to PVDF membranes. 15 µg of protein lysate was loaded for human fibroblast samples while 30 µg of protein was loaded for mouse hypothalamus samples. PVDF membrane blots were incubated in blocking buffer (5%, w/v, dried skimmed milk in Tris-buffered saline, pH 7.4, and 0.2% Tween 20, TBST) for 1 hr at room temperature, followed by overnight incubation at 4 °C with primary antibodies diluted in blocking buffer. Blots were then rinsed three times 7 minutes each with TBST and incubated in HRP conjugated secondary antibody diluted in blocking buffer for an additional 75 minutes at room temperature. Following secondary antibody incubation, the blots were washed four times 7 minutes each in TBST and imaged using SuperSignal West Femto Maximum Sensitivity Substrate (Thermo Fisher Scientific) and an ImageQuant LAS 4000 (GE) imager. Antibody information is detailed in Supplementary Table 3. Uncut blots are shown in Supplementary Fig. 5.

### Immunofluorescence

Human fibroblasts were grown on glass coverslips in 24-well plates, while iNeurons were grown on 8-well glass slides (MilliporeSigma). Cells were rinsed once with PBS and fixed with 10% Formalin for 12 minutes or 4% PFA for 20 minutes at room temperature. Cells were rinsed three times with PBS, permeabilized with 0.1% Triton X-100 in 1X PBS for 10 minutes, and blocked with blocking reagent (10% donkey serum in PBS) for 30 minutes at room temperature. Cells were then incubated with primary antibodies diluted in blocking buffer overnight at 4 °C. The next day cells were washed three times with PBS and then incubated with secondary antibodies diluted in blocking buffer for an additional 60 minutes in the dark, at room temperature. Nuclei were stained with 1 ng/mL DAPI for 20 minutes in the dark. Cells were then rinsed four times with PBS and mounted on glass slides using Mowiol mounting media.

Human fibroblasts transfected with P62-GFP-RFP or LC3-GFP were grown on glass coverslips in 24 well plates. Prior to imaging, cells were rinsed once with PBS, fixed with 10% formalin for 12 minutes at room temperature, rinsed with PBS again, and mounted on glass slides using Mowiol mounting media.

### Oil red O staining

Human fibroblasts were grown to confluency in 6-well tissue culture treated dishes. Upon reaching confluency, the cells were grown for 8 additional days with a media change every other day. Three days prior to harvest, cells were treated with DMSO or 100 nM rapamycin for 72 hours. At the end of the growth period, the cells were rinsed once with PBS and fixed with 10% formalin for 30 minutes at room temperature. To prepare the Oil Red O working solution, Oil Red O stock solution (0.5% Oil Red O in isopropanol – Sigma) was diluted 3:2 in diH_2_O. Working solution was allowed to sit for 10 minutes after mixing, and was then filtered through a 0.2 µm syringe filter. Working solution was prepared fresh for each experiment. After formalin fixation, cells were rinsed twice with diH_2_O and incubated 5 minutes in 60% isopropanol. Cells were then incubated in Oil Red O working solution for 20 minutes, and rinsed five times with diH_2_O. Staining was visualized under a brightfield microscope. For quantification, lipid droplets were lysed to release Oil Red O stain by adding 1 mL 100% isopropanol per well and incubating with rocking for 5 minutes. Isopropanol was collected from each well, and absorbance was measured at 492 nm.

### Neuronal analysis

Z-stack confocal images, taken at 1 µm intervals at 20x magnification, were deconvoluted and imported to Neurolucida for tracing and analysis. After user guided tracing of dendrites was performed, Sholl analysis and generation of dendritic morphology data was performed in Neurolucida using batch analysis for each group.

### Statistics

Prism and Microsoft Excel were used to carry out all statistical analyses. Students T-test was performed for all statistics comparisons of two experimental groups (e.g. control vs. SHFYNG). For all statistical comparisons of three or more groups, a one-way ANOVA with Tukey correction for multiple comparisons was performed.

## Supplementary information

Supplementary Material

## Data Availability

The datasets generated during and/or analysed during the current study are available from the corresponding author on reasonable request.
