# Supplementary material for: mTOR and autophagy pathways are dysregulated in murine and human models of Schaaf-Yang syndrome
Source: Sci Rep. 2019 Nov 4;9:15935. doi: 10.1038/s41598-019-52287-2 (PMC6828689; doi:10.1038/s41598-019-52287-2)

Supplemental Material

mTOR and autophagy pathways are dysregulated in murine and human models of Schaaf-Yang syndrome

Emeline Crutcher[1,2,3], Rituraj Pal[2,3], Fatemeh Naini[2,4], Ping Zhang[5,6], Magdalena Laugsch[7], Jean Kim[5,6], Aleksandar Bajic[2,4], Christian P. Schaaf[1,2,3,7*]

Supplementary Table 1. Human cell line (fibroblasts and iPSCs) information.

Line	Mutation	Inheritance	Age at biopsy	Gender	Fibroblast	iPSC/# of clones
Control 1	none		30	Male	✔	
Control 2	none		N/A	N/A	✔	
Control 3	none		10	Male	✔	✔/2
Control 4	none		22	Male		✔/2
SHFYNG 1	c.1996dupC	paternal	15	Male	✔	✔/2
SHFYNG 2	c.1996dupC	de novo	1	Male	✔	✔/1
SHFYNG 3	c.1912C>T	de novo	10	Male	✔	

Shading indicates cells intentionally left blank. Control 3 and SHFYNG 1 and 2 are individuals from which both fibroblast and iPSC lines were used.

Supplementary Table 2. qRT-PCR primer sequences

Primer	Sequence
h_mTOR_F	GCCGACTCAGTAGCAT
h_mTOR_R	CGGGCACTCTGCTCTTT
h_IPW116_F	CTGGTGGATCCCACAGGT
h_IPW116_R	AGAAGCCCACGCCACATA
h_OCT4_F	AGAACATGTGTAAGCTGCGG
h_OCT4_R	GTTGCCTCTCACTCGGTTC
h_NANOG_F	TTGTGGGCCTGAAGAAAACT
h_NANOG_R	ATCTGCTGGAGGCTGAGGTA
h_VGLUT2_F	ATTCCATCAGCAGCCAGAGT
h_VGLUT2_R	AGGAGGTGGTTGCCAGTCTA
h_NCAM_F	GACATCACCTGCTACTTCCTG
h_NCAM_R	GGCTCCTTGGACTCATCTTTC
h_β-Actin_F	GGACTTCGAGCAAGAGATGG
h_β-Actin_R	AGCACTGTGTTGGCGTACAG
h_GUSB_F	AAACGATTGCAGGGTTTCAC
h_GUSB_R	CTCTCGTCGGTGACTGTTCA
m_mTor_F	CATTGGCTGGTGTCCCTTCT
m_mTor_R	TCTCTTAGCCATGTTGGCCC
m_HG116_F	AAGTGTCACCACAACACTGGAC
m_HG116_R	AAGCTGCTGGTAGAAGAAATGG
m_Gapdh_F	CAAGGAGTAAGAAACCCTGGACC
m_Gapdh_R	CGAGTTGGGATAGGGCCTCT

Supplementary Table 3. Primary antibody information

Antibody	Catalog #	Supplier	Dilution
P-mTOR	2971S	Cell Signaling	1:500
mTOR	2972S	Cell Signaling	1:500
P-S6K1	9234S	Cell Signaling	1:500
S6K1	9202S	Cell Signaling	1:500
P-S6	2215S	Cell Signaling	1:1,000
S6	2217S	Cell Signaling	1:1,000
P-ULK1	6888S	Cell Signaling	1:500
ULK1	8054S	Cell Signaling	1:500
P-62 (human)	610833	BD Transduction Laboratories	1:1,000
P-62 (mouse)	H00008878-M01	Abnova	1:1,000
LC3	2775S	Cell Signaling	1:500
GAPDH	CB1001	Millipore-Sigma	1:10,000
β-Actin	JLA20	DSHB Iowa	1:1,000
Vinculin	V9131	Sigma	1:10,000
GFP	ab6556	Abcam	1:10,000
OCT4	560186	BD Biosciences	1:40
SOX2	ab97959	Abcam	1:200
SSEA4	FAB1435A	R&D Systems	1:10
β-III-Tubulin	MAB1637	Millipore-Sigma	1:200
P-4EBP1	2855S	Cell Signaling	1:1000
4EBP1	9452S	Cell Signaling	1:1000
P-AMPK	2531S	Cell Signaling	1:1000
AMPK	2532S	Cell Signaling	1:1000

Supplementary Figure 1

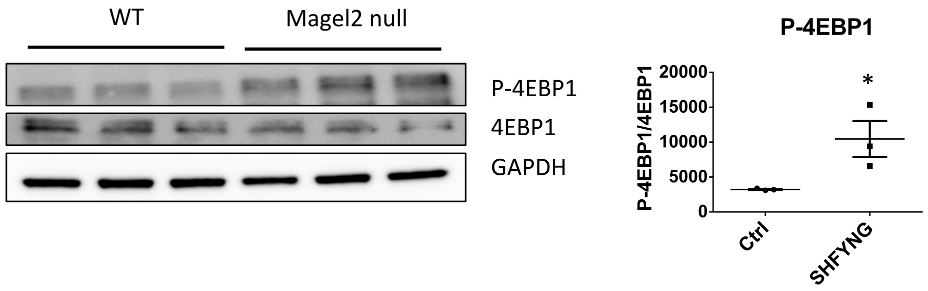

Supplementary Figure 1. Increased expression of mTORC1 target P-4EBP1 in Magel2 null mouse hypothalamus. * = p<0.05 by Student's T-test

Supplementary Figure 2

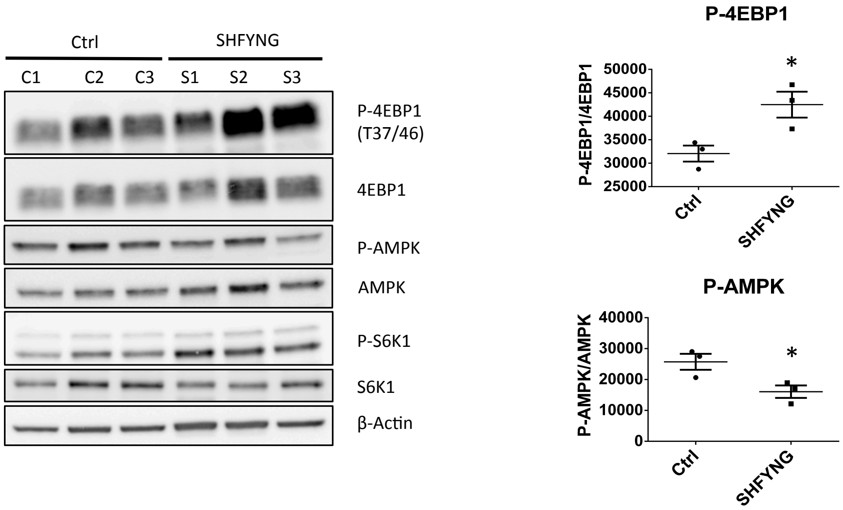

Supplementary Figure 2. mTORC1 target P-4EBP1 is upregulated, mTORC1 negative regulator P-AMPK is downregulated in SHFYNG patient fibroblasts. * = $p<0.05$ by Student's T-test

Supplementary Figure 3

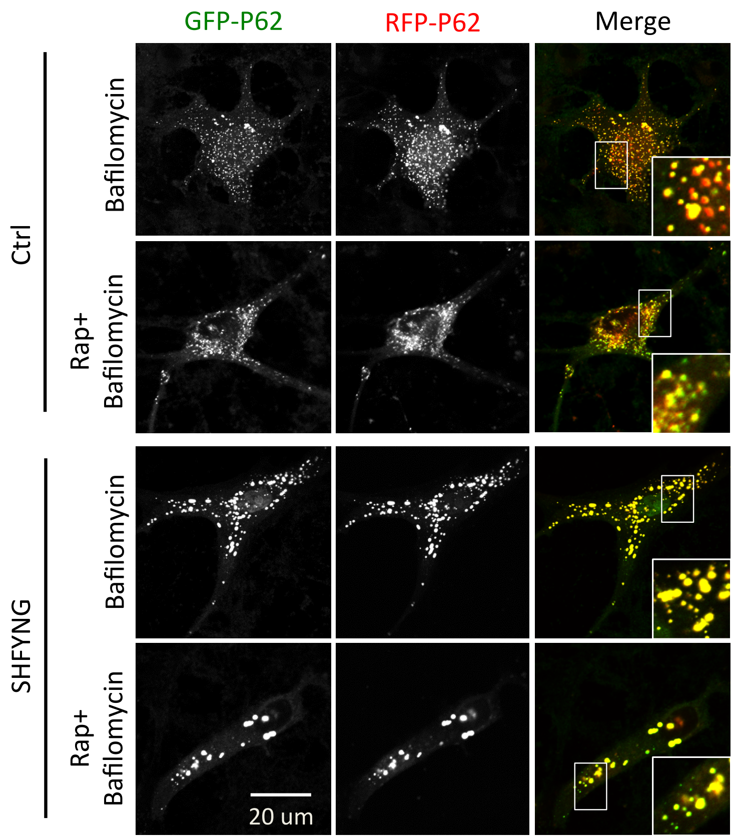

Supplementary Figure 3. Bafilomycin treatment blocks autophagy in rapamycin treated and untreated SHFYNG patient and control lines.

Supplementary Figure 4

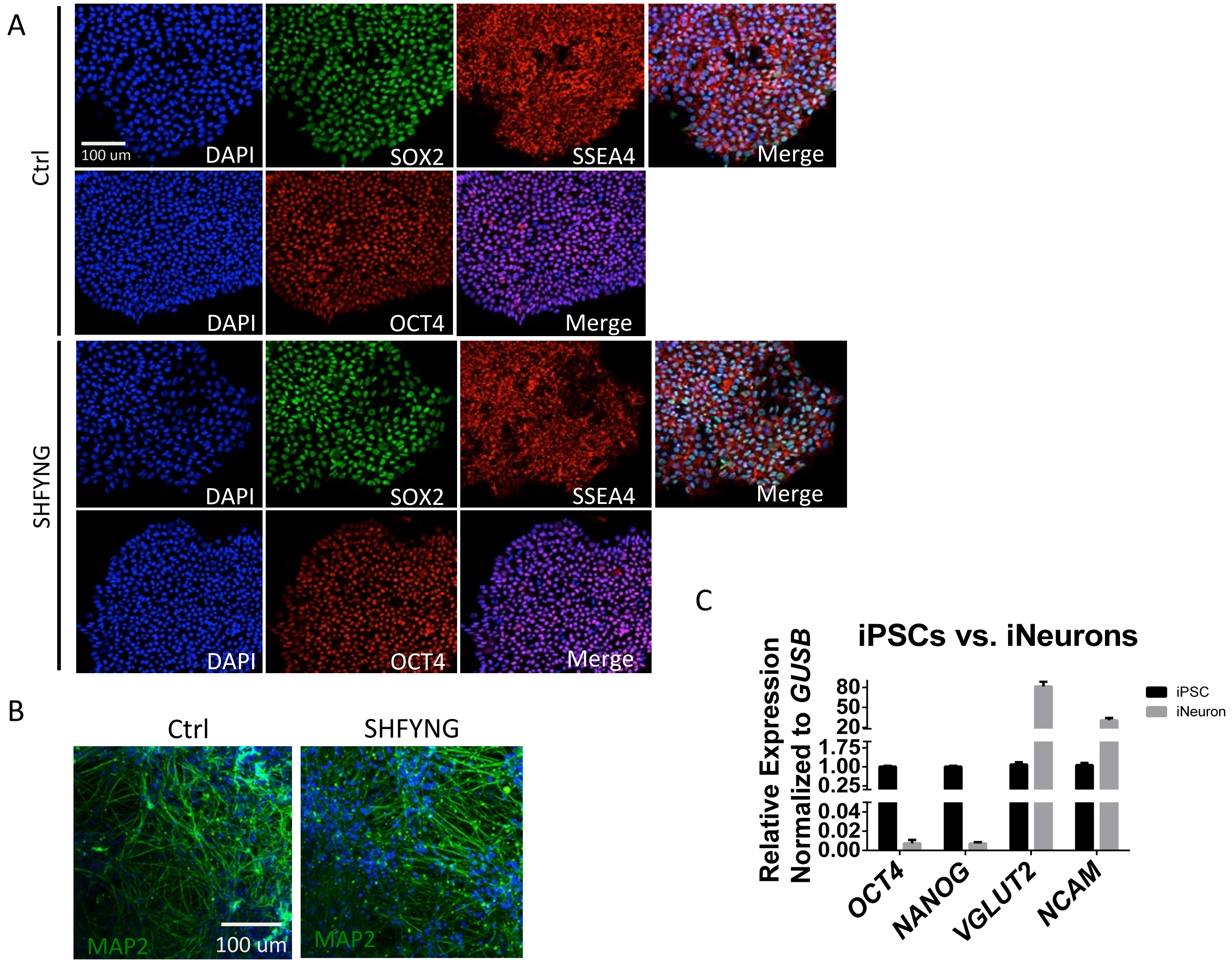

Supplementary Figure 4. A. Representative immunofluorescence images showing pluripotency marker expression in iPSC lines from SHFYNG patients and controls. B. Representative immunofluorescence images showing β-III-tubulin staining in D30 SHFYNG patient and control iPSC derived neurons. C. Transcript level of pluripotent and neuronal genes between iPSC and D30 iNeuron states. Relative expression levels of transcripts in the iPSC state are set to 1. All control and SHFYNG patient lines were pooled and included in this data set.

Supplementary Figure 5: Uncut blots

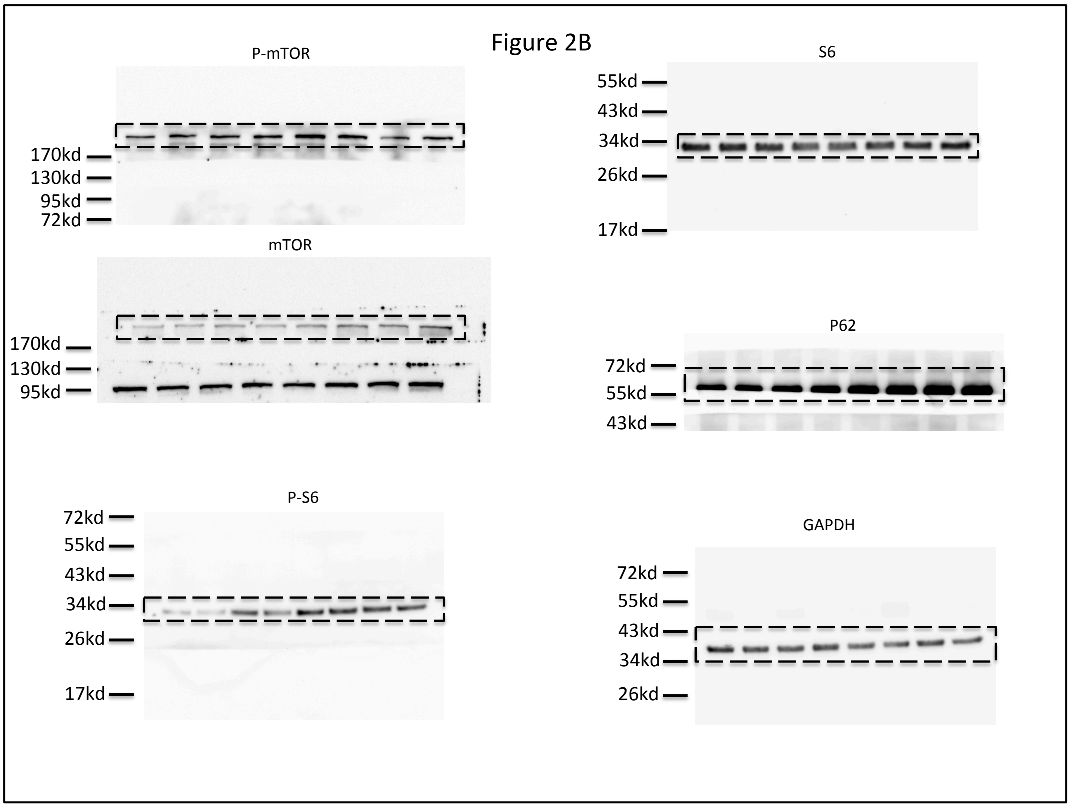

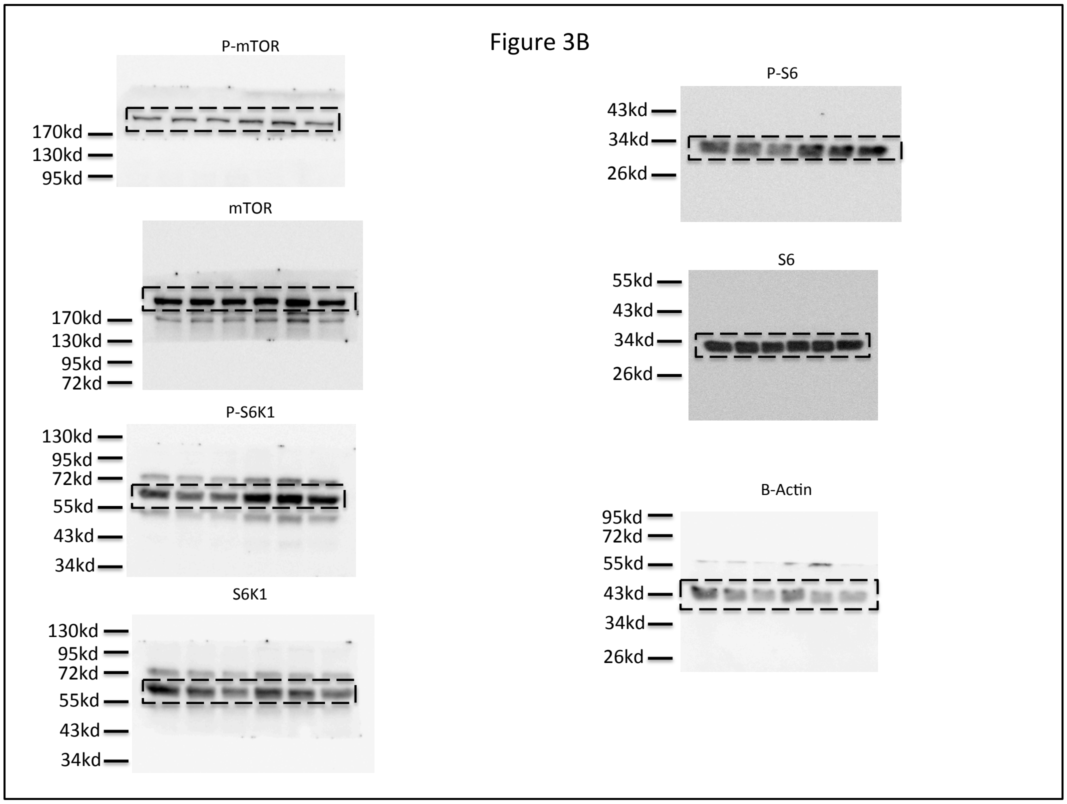

Supplementary Figure 5: Uncut blots (Cont.)

Figure 3C

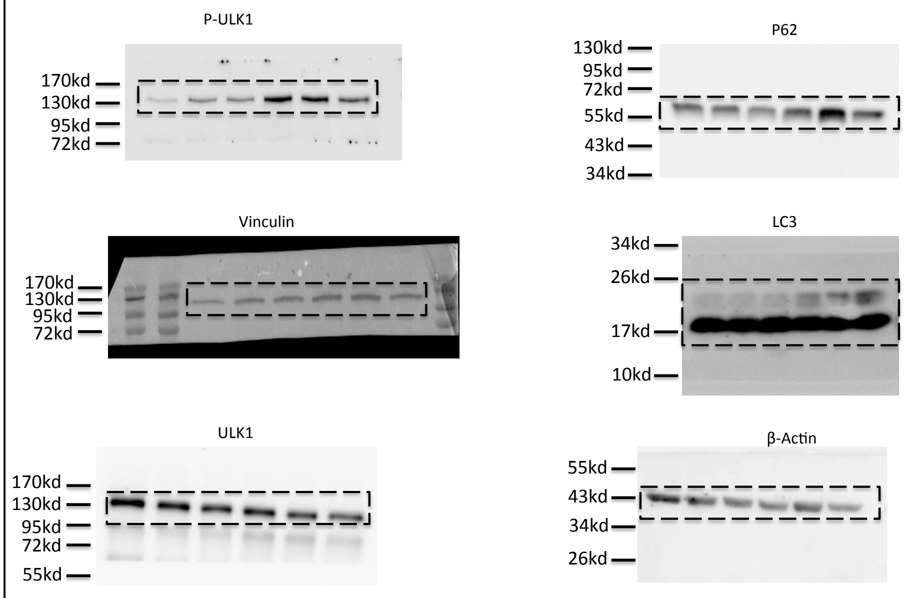

Figure 4A

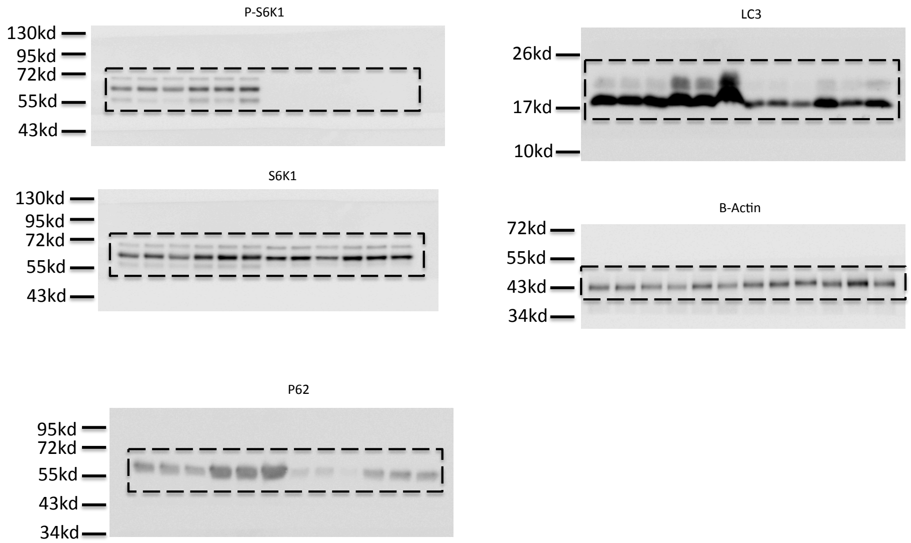

Supplement: Supplementary file 1 — Supplementary Material [file 41598_2019_52287_MOESM1_ESM.pdf]
